# SIRT3 overexpression and epigenetic silencing of catalase regulate ROS accumulation in CLL cells activating AXL signaling axis

**DOI:** 10.1038/s41408-021-00484-6

**Published:** 2021-05-17

**Authors:** Guru P. Maiti, Sutapa Sinha, Hasan Mahmud, Justin Boysen, Mariana T. Mendez, Sara K. Vesely, Jennifer Holter-Chakrabarty, Neil E. Kay, Asish K. Ghosh

**Affiliations:** 1 Stephenson Cancer Center, University of Oklahoma Health Sciences Center, Oklahoma City, OK 73104 USA; 2 Division of Hematology, Mayo Clinic, 200 First Street SW, Rochester, MN 55905 USA; 3 Department of Pathology, University of Oklahoma Health Sciences Center, Oklahoma City, OK 73104 USA; 4 Hudson College of Public Health, University of Oklahoma Health Sciences Center, Oklahoma City, OK 73104 USA

**Keywords:** Chronic lymphocytic leukaemia, Cell signalling

## Abstract

Mitochondrial metabolism is the key source for abundant ROS in chronic lymphocytic leukemia (CLL) cells. Here, we detected significantly lower superoxide anion (O_2_^−^) levels with increased accumulation of hydrogen peroxide (H_2_O_2_) in CLL cells vs. normal B-cells. Further analysis indicated that mitochondrial superoxide dismutase (SOD)2, which converts O_2_^−^ into H_2_O_2_ remained deacetylated in CLL cells due to SIRT3 overexpression resulting its constitutive activation. In addition, catalase expression was also reduced in CLL cells suggesting impairment of H_2_O_2_-conversion into water and O_2_ which may cause H_2_O_2_-accumulation. Importantly, we identified two CpG-islands in the catalase promoter and discovered that while the distal CpG-island (−3619 to −3765) remained methylated in both normal B-cells and CLL cells, variable degrees of methylation were discernible in the proximal CpG-island (−174 to −332) only in CLL cells. Finally, treatment of CLL cells with a demethylating agent increased catalase mRNA levels. Functionally, ROS accumulation in CLL cells activated the AXL survival axis while upregulated SIRT3, suggesting that CLL cells rapidly remove highly reactive O_2_^−^ to avoid its cytotoxic effect but maintain increased H_2_O_2_-level to promote cell survival. Therefore, abrogation of aberrantly activated cell survival pathways using antioxidants can be an effective intervention in CLL therapy in combination with conventional agents.

## Introduction

CLL is an adult B-cell malignancy with highly variable disease course^1^ and unpredictable response to therapeutic agents. Reactive oxygen species (ROS) have been identified as signaling molecules in various pathways regulating cell survival. Oxidants such as H_2_O_2_ are connected to lymphocyte activation while the molecular mechanisms are less clear^2^.

The main physiological function of mitochondria is the production of ATP by oxidative phosphorylation and the essential metabolites to accomplish the bioenergetics and biosynthetic demands of cells^3^. A fundamental observation in biology is that mitochondrial function, as measured by increased ROS, changes with age, suggesting a potential mechanistic link between the cellular processes governing longevity and mitochondrial metabolism homeostasis. Thus, mitochondrial dysfunction inevitably enhances ROS production resulting in oxidative stress, which are observed in multiple age-related illnesses including cancer^4^. Increased ROS levels promote genetic instability and development of drug resistance, harness cell signaling^5,6^, and altogether account for cancer cells’ aggressive behavior^7^. Thus, a challenge for novel therapeutic strategies will be the fine tuning of intracellular ROS signaling to effectively deprive cells from ROS-induced tumor promoting events, towards tipping the balance to ROS-induced apoptotic signaling or ROS inhibition using antioxidants.

PI3K/AKT activation via ligation of growth factors to their cellular receptors is associated with increased oxygen consumption as well as an increase in total cellular ATP derived from both glycolytic and oxidative sources^8,9^ in mitochondria resulting in accumulation of ROS^9^ while other factors may also play a role^10,11^. Among the mitochondrial Sirtuins, SIRT3 with NAD^+^-dependent protein deacetylase activity^12^ regulates the production of ROS via the electron transport chain, as well as the detoxification of ROS through activation of antioxidant enzymes. SODs are a class of enzymes that catalyze the detoxification of O_2_^−^ into O_2_ and H_2_O_2,_ followed by conversion of H_2_O_2_ into O_2_ and water by catalase. Mitochondrial SOD2 is an antioxidant enzyme and plays a crucial role in controlling ROS production. SIRT3 lowers ROS levels by deacetylating and increasing enzymatic activity of SOD2^13,14^. Elevated ROS, on the other hand, activates SIRT3 expression to deacetylate SOD2 and thus clearing ROS^15^. Furthermore, SIRT3 interacts with Forkhead box O (FOXO)3a transcription factor to activate SOD2 and catalase gene transcription^16^, suggesting the existence of a functional interplay between SIRT3/SOD2/catalase and accumulation of ROS.

Cancer cells including leukemic cells live under oxidative stress, which may lead to cell proliferation and transformation. Several studies have identified mitochondrial metabolism as the key source for ROS accumulation in CLL which, on the other hand, is linked to CLL cell survival^17–19^. However, the precise mechanism of ROS elevation and activation of survival pathway(s) in CLL remain largely undefined. In this study, we have shown that overexpression of SIRT3 and variable degrees of promoter methylation of the catalase gene may impair ROS clearance leading to accumulation of H_2_O_2_ in CLL cells which, on the other hand, activates the AXL/AKT/ERK and fibroblast growth factor receptor (FGFR) cell survival signaling axes. Of relevance, earlier, we have shown that CLL cells express AXL as a constitutively active receptor tyrosine kinase (RTK), albeit at differential levels, and acts as a docking site of multiple signal mediators including PI3K/AKT, Lyn, SYK/ZAP70 and phospholipase C (PLC)γ2, regulating CLL cell survival^20,21^. In addition, AXL acts as an upstream regulator of FGFR signal in CLL cells ^22^.

## Materials and methods

### Reagents

Phospho-tyrosine antibody 4G10 (Millipore); Actin, AXL, β-tubulin, GAPDH antibodies (Santa Cruz Biotechnologies) and fluorescence-conjugated antibodies to CD5 and CD19 (BD Biosciences) were used in this study. All other antibodies were obtained from Cell Signaling Technologies. Dihydroethidium (DHE) and 2′,7′-dichlorodihydrofluorescein diacetate (CM-H2DCFDA) (Molecular Probes); EpiMark Bisulfite conversion Kit (New England BioLabs); IL-2/IL-15 (Peprotech); demethylating agent, 5’-aza-deoxycytidine (AZA), RNA isolation kit, H_2_O_2_, Trolox (Sigma); mitochondria isolation kit (ThermoScientific); AXL-inhibitor SGI-7079 (Selleckchem); catalase and GAPDH primers (SABiosciences) were also purchased. CpG oligodeoxynucleotides (ODN) were synthesized in the Mayo Clinic Genomic Core facility.

### Sample size and isolation/purification of primary cells

Blood samples from a total of 67 previously untreated CLL patients and 30 age-matched, normal healthy subjects were obtained for this study. Samples from normal individuals (N) or CLL patients (P) were assigned arbitrary numbers and presented in Supplementary Table S1. The mean age for the 67 CLL patients was 67.7 years (standard deviation of 10.0, range 44–91 years). Among the CLL patients 73% (49/67) were male, 22% (14/65) were Rai Stage III or IV, 63% (29/46) had IGHV mutation, 30% (15/50) ZAP70 positive (≥20%), 27% (16/60) CD38 positive ((≥20%), and 23% (14/61) normal FISH. CLL samples were randomly selected for each experiment based on cell availability but not based on any patient characteristics or experiment outcomes. All patients provided written informed consent according to the Declaration of Helsinki to the OUHSC and Mayo Clinic Institutional Review Boards, which approved these studies.

Primary CLL cells or normal B-cells were purified from blood of CLL patients or healthy individuals, respectively, using RosetteSep B-cell enrichment kit (STEMCELL)^20,21^. Typical purification range of CD5^+^/CD19^+^ CLL cells was ≥95–99% and >95% pure CD19^+^ B-cells were obtained from normal blood.

### Cell culture

CLL cells were cultured for optimum viability in serum-free AIM-V (GIBCO) medium^20,21^. A CLL-derived cell line (MEC1), diffuse large B-cell lymphoma cell lines (DHL2, DHL6, and LY3) and a mantle cell lymphoma cell line (Mino) were purchased from ATCC. Cell lines were tested for *Mycoplasma* negativity before use.

### Measurement of H_2_O_2_ and O_2_^−^

1–2 × 10^6^ purified, normal B-cells or CLL cells were incubated with CM-H_2_DCFDA (10 μM) to detect H_2_O_2_ or DHE (5 µM) for O_2_^−^ at 37 °C for 1 h, followed by flow cytometric analysis (BD Canto II).

### Immunoprecipitation and western blot analysis

Tyrosine-phosphorylated proteins or AXL were immunoprecipitated from CLL cell lysates (0.2 mg) using 4G10 or an AXL-specific antibody, respectively, followed by western blots^20,21^ to detect total tyrosine-phosphorylated proteins or P-AXL using 4G10 antibody.

### Preparation of mitochondrial fraction

Mitochondrial/cytoplasmic fractions were isolated from 5 × 10^7^ normal B-cells or CLL cells using a mitochondrial isolation kit. Equal amount of mitochondrial fractions were analyzed to detect comparative levels of acetylated-SOD2/total SOD2 in western blots using specific antibodies.

### Reverse transcription polymerase chain reaction

Total RNA (1 μg) extracted from normal B-cells or CLL cells was reverse transcribed using the SuperScript^®^ III First Strand Synthesis Kit (Invitrogen) and qPCR was performed in triplicate for catalase expression using specific primers and the SYBR Green PCR core Master Mix (Applied Biosystems). GAPDH was used for cDNA normalization. Relative expression was calculated using the comparative Ct method and presented as “fold expression”.

### Methylation study

Promoter methylation status of the catalase gene was analyzed by PCR-based methylation-specific PCR (MSP) after bisulfite modification of genomic DNA using a bisulfite conversion Kit. All the primers (Supplementary Table S2) were designed using ‘Meth Primer’ online primer designing tool^23^. Briefly, 1 µg of genomic DNA isolated from CLL cells or normal B-cells was modified by bisulfite reaction, followed by PCR using methylation/unmethylation-specific (M/U) primer sets. PCR products were analyzed on agarose gels.

CG-methylation status of the catalase promoter was further analyzed by sequencing the PCR products of the bisulfite-converted genomic DNA from normal B-cells (*n* = 5), CLL cells (*n* = 32), and B-cell lymphoma cell lines. Briefly, PCR products were purified from gels, sequenced (OUHSC DNA Sequencing facility) and scored for methylation in each ‘CG’ site as one allele or both allele or no methylation at both the alleles depending on the presence of both C/T peak or only C peak or only T peak in chromatogram, respectively. Catalase promoter methylation status of individual samples was calculated as percent (%) of methylated CG sites.

### Treatment of cells with 5’-azacitidine and BCR stimulation

MEC1/DHL6 cells (0.5 × 10^6^ cells/mL) were treated with 1 µM AZA for 72 h, followed by release of treatment for 24 h or left untreated and total RNA was extracted. Similarly, primary CLL cells were treated with 2.5 µg/mL CpG ODN, 100 U/mL IL-2, and 10 ng/mL IL-15 with 1 µM AZA for 72 h or left untreated^24^ for RNA extraction. Catalase mRNA levels were determined by qRT-PCR.

For BCR stimulation, purified CLL cells (5.0 × 10^6^/mL) from treatment naïve CLL patients (P65 and P68) were treated in vitro with 10 μg/mL goat anti-human IgM antibody for 0, 15, 30, and 60 min or left untreated. Cell lysates were analyzed for the phosphorylation of BTK and expression of SIRT3 in western blots using specific antibodies.

### Treatment of CLL cells with H_2_O_2_ and inhibitors of ROS, AXL, or BTK

CLL cells (5 × 10^6^/mL) pretreated with DMSO or sublethal dose of a high-affinity AXL inhibitor, SGI-7079^25^ or a BTK inhibitor, ibrutinib (0.75 µM for 1 h) were exposed to H_2_O_2_ (0.6 mM) for 5 min^26^ or left untreated/unexposed. Cell lysates were prepared as described earlier^20–22^ to analyze the activation status of AXL, BTK, AKT, ERK1/2, and expression of SIRT3/SOD2 in immunoprecipitation/western blots using specific antibodies.

In few experiments, CLL cells were also treated with 50–100 µM of H_2_O_2_ or a ROS-inhibitor, Trolox (50 µM) for 4 h, and cell lysates were analyzed for P-AKT/P-ERK1/2 and SIRT3 expression in western blots.

### Statistical analysis

**Fig. 1:**
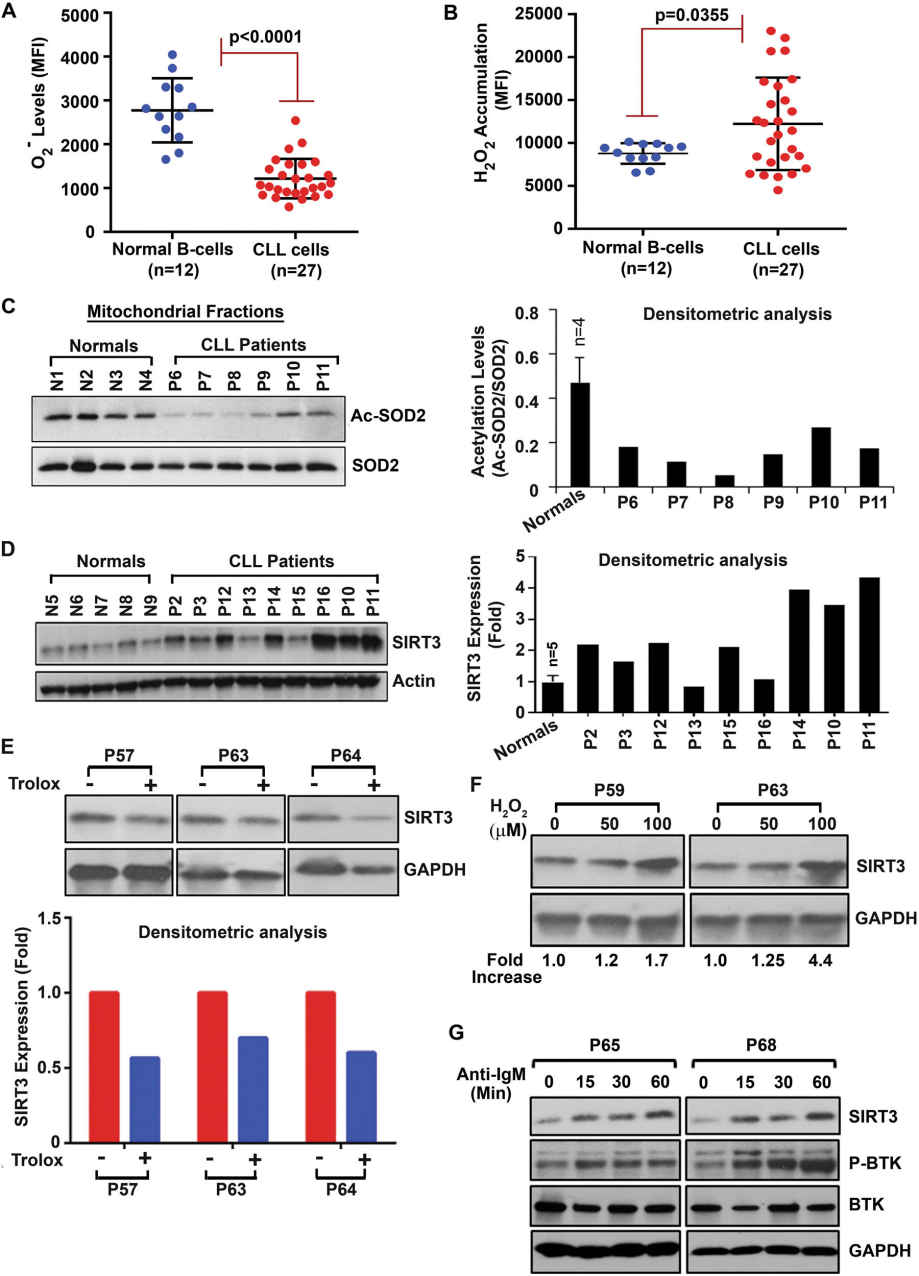
Deregulation of the SIRT3/SOD2 axis in CLL cells influences ROS generation. **A**, **B** CLL cells contain lower levels of O_2_^−^ but higher levels of H_2_O_2_. Purified CLL cells or normal B-cells were stained with DHE to detect O_2_^−^ levels (**A**) or DCFDA to detect accumulation of H_2_O_2_ (**B**) and analyzed by flow cytometry. Results are presented as mean fluorescent intensity (MFI) ± one standard deviation. **C** SOD2 remains as constitutively active. Mitochondrial fractions isolated from purified CLL cells or normal B-cells (N1–N4) (obtained from panels **A/B**) were analyzed for acetylated-SOD2 (Ac-SOD2 [K68]) in western blot using a specific antibody. Total mitochondrial SOD2 was used as loading control. Densitometric analysis of the blots was also performed to determine relative levels of Ac-SOD2 (right panel). **D** CLL cells overexpress SIRT3. Purified normal B-cells from healthy subjects (N5–N9) or CLL cells (P2, P3, P10–P16) were analyzed for the expression of SIRT3 in western blot using a specific antibody. Actin was used as loading control. Densitometric analysis was performed to show relative change. **E** ROS inhibition reduces SIRT3 level. Purified CLL cells (P57, P63, P64) treated with a ROS-inhibitor Trolox (50 µM/4 h) or left untreated were analyzed for SIRT3 expression as in panel **D**. GAPDH was used as loading control. Densitometric analysis was performed to show relative change (lower panel). **F** ROS activates SIRT3 expression. Purified CLL cells (P59, P63) treated in vitro with H_2_O_2_ (50 µM and 100 µM) for 4 h were analyzed for SIRT3 expression as described in panel **E**. Densitometric analysis was performed to show relative fold-increase of SIRT3. **G** BCR activation induces SIRT3 expression. Purified CLL cells (P65, P68) treated with an anti-IgM antibody for the indicated time periods were analyzed for the expression of SIRT3 and P-BTK levels (as an indicator of BCR activation) in western blots using specific antibodies. BTK and GAPDH were used as loading controls.

A Wilcoxon test was calculated to compare the values of O_2_^−^ levels (MFI) between normal B-cells and CLL cells since the data was not normally distributed. A two-tailed independent *t*-test for unequal variances (Satterthwaite) was calculated to compare the H_2_O_2_ accumulation between normal B-cells and CLL cells. The Spearman’s correlation test was calculated to evaluate the degree of CpG methylation in Island-I of the catalase promoter and its protein levels in CLL cells. PRIZM GraphPad software was used to create the statistical figures and for statistical analysis with the exception of the Spearman correlation coefficient that was calculated using SAS 9.4 (Cary, NC). With 12 normal individuals and 27 CLL patients in ROS measurement group (Fig. 1, panels A, B), an independent *t*-test will have at least 80% power at the 5% significance level to detect an effect size of 1.0. Experiments were repeated for three times and presented the representative results, as appropriate.

## Results

### CLL cells generate increased levels of H_2_O_2_ with reduced O_2_^−^ levels

Two of the most important ROS components generated in cells are O_2_^−^ and H_2_O_2_. Thus, in an effort to assess the levels of O_2_^−^ and H_2_O_2_, freshly isolated CLL cells (*n* = 27) and normal B-cells (*n* = 12) were treated with DHE or DCFDA to detect O_2_^−^ and H_2_O_2_, respectively, by flow cytometry. While we detected significantly lower O_2_^−^ levels (*p* < 0.0001) (Fig. 1A), increased accumulation of H_2_O_2_ (*p* = 0.0035) was discernible, albeit in variable levels, in CLL cells (Fig. 1B) from the majority of CLL patients compared to normal B-cells under similar experimental conditions. Further analysis indicated that the levels of H_2_O_2_ accumulated in CLL cells were not associated with the known CLL prognostic factors including IGHV mutational status or ZAP70 positivity, although the sample size was small. Together, these findings suggest that compared to normal B-cells, (i) mitochondrial SOD2 may be highly active in CLL cells; while (ii) conversion of H_2_O_2_ into O_2_ and water is likely impaired.

### Mitochondrial SOD2 remains highly active in CLL cells

SOD2 is reported to have a crucial role in controlling the level of ROS, as mitochondria consume over 90% of intracellular oxygen and generate a large flux of ROS^15^. Thus, to delineate the mechanism of why CLL cells generate lower levels of O_2_^−^, we first assessed the mitochondrial SOD2 activation status. While oxidative stress-induced SOD2 expression is believed to be an important cellular defense mechanism^27^, acetylation/deacetylation at the lysine-68 residue (K68) of SOD2 regulates its activity^15^. Therefore, to define the acetylation status as a readout of SOD2 activation in CLL cells, mitochondrial fractions were isolated from purified CLL cells and normal B-cells. First, the expression of voltage-dependent anion channel (VDAC) protein, ubiquitously expressed and located in the outer mitochondrial membrane^28^, was analyzed to assess the purity of the cytoplasmic/mitochondrial fractions isolated from purified normal B-cells (N1–N4) or CLL cells (P6, P10, and P11) in western blots using a specific antibody. Results suggested that the mitochondrial fractions used in these experiments were highly purified based on the presence of VDAC, while it was not detectable in the cytoplasmic fractions (Supplementary Fig. S1). Further analysis detected that the levels of acetylated-SOD2 at K68 were substantially reduced in the mitochondrial fractions of CLL cells as compared to that in normal B-cells (Fig. 1C), suggesting that SOD2 in CLL cells is likely enzymatically highly active converting O_2_^−^ into H_2_O_2_ more efficiently. Importantly, we did not find any significant alteration of SOD2 levels between CLL cells and normal B-cells (Fig. 1C). Together, these findings may explain, at least in part, why CLL cells exhibiting lower levels of O_2_^−^ compared to normal B-cells (Fig. 1A).

### CLL cells overexpress SIRT3

As SIRT3 is known to deacetylate and activate SOD2^15^, we next assessed SIRT3 expression status in CLL cells by western blots. Indeed, compared to normal B-cells, we detected CLL cells overexpressed SIRT3, albeit at variable levels (Fig. 1D). Of relevance, earlier studies suggested that pharmacological augmentation of mitochondrial ROS increases SIRT3 mRNA and protein levels in in vitro cell culture system^15,29^. Thus, we examined if ROS regulates SIRT3 levels in CLL cells. Indeed, inhibition of endogenous ROS in CLL cells reduced SIRT3 expression (Fig. 1E) while, in vitro treatment of leukemic B-cells with exogenous H_2_O_2_, which generates ROS^30^ upregulated SIRT3 (Fig. 1F). Together, these findings indicate the existence of a positive feedback loop between ROS accumulation and SIRT3 expression in CLL cells.

Engagement of the B-cell receptor (BCR) in B-lymphocytes initiates a series of tightly controlled signaling events including activation of AKT, ERK1/2, and NF-κB pathways leading to transcription of genes responsible for B-cell development, survival and proliferation. As BCR signaling pathway has emerged as a key driver for the expansion of neoplastic B-cell clones and pathogenesis in several B-cell malignancies including CLL^31^, we explored if in vitro activation of BCR signal in primary CLL cells could also induce SIRT3 expression. Indeed, we detected, enforced BCR-activation-induced SIRT3 expression in CLL cells (as early as in 15 min) in a time-dependent manner (Fig. 1G). In total, our findings suggest that while BCR signal upregulates SIRT3, elevation of ROS may maintain its constitutive level in CLL cells.

### CLL cells express reduced levels of catalase

**Fig. 2:**
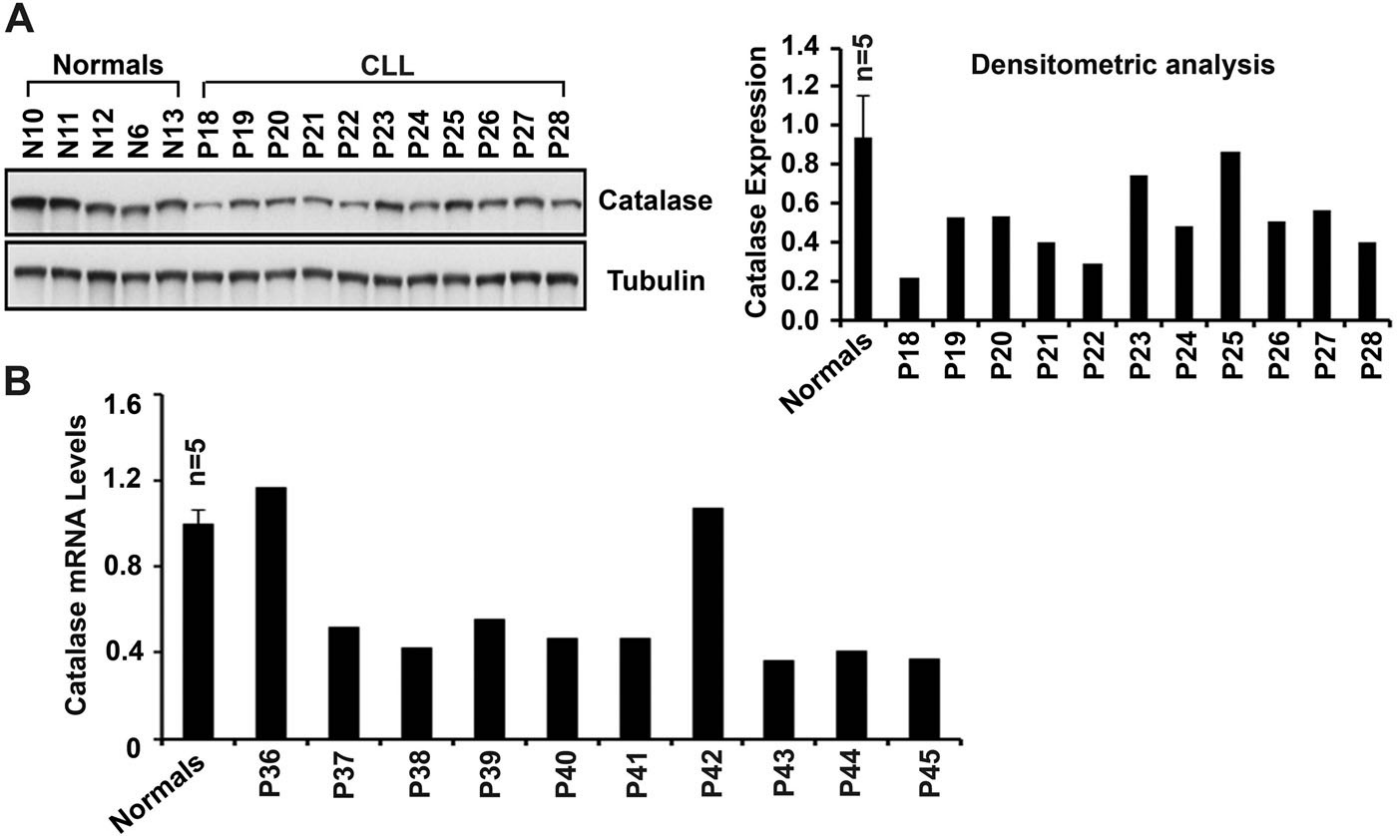
CLL cells express reduced levels of catalase. **A** Purified normal B-cells from healthy individuals (*n* = 5; N6, N10–N13) or CLL cells from CLL patients (*n* = 11; P18–P28) were analyzed for the expression of catalase in western blot using a specific antibody. β-tubulin was used as loading control. Densitometric analysis of the blot was also performed to determine relative levels of catalase protein (right panel) with respect to the mean normalized value of catalase levels in normal B-cells. **B** Total RNA from purified normal B-cells (*n* = 5; N6, N10–N13) or CLL cells purified from the indicated CLL patients (*n* = 10; P36–P45) was reverse transcribed and quantitative PCR for catalase was performed in triplicate using the SYBR Green PCR core Master Mix. GAPDH was used for cDNA normalization. Relative transcript levels of catalase was calculated using the comparative Ct method.

While we detected lower levels of O_2_^−^, an increased accumulation of H_2_O_2_ was also detected in CLL cells (Fig. 1B); suggesting that conversion of H_2_O_2_ into water and O_2_ is likely to be impaired. As catalase enzyme catalyzes the conversion process of H_2_O_2_, its expression level is a critical factor for ROS clearance from the cells. Therefore, to define why CLL cells from some patients show increased H_2_O_2_ accumulation, catalase protein levels were assessed in CLL cells vs. normal B-cells in western blots. Indeed, reduced expression of catalase was detectable in CLL cells from majority of patients as compared to normal B-cells (Fig. 2A).

Next, to elucidate if reduced protein levels of catalase in CLL cells was a result of its reduced mRNA expression, we quantified catalase transcript levels in CLL cells from randomly chosen patients (*n* = 10) by qRT-PCR. Consistent with the catalase protein levels, we also detected reduced levels of catalase transcripts in CLL cells from majority of CLL patients (8 of 10) as compared to the mean value of catalase mRNA levels in normal B-cells (*n* = 5) (Fig. 2B); suggesting that catalase expression in CLL cells is likely regulated at the level of transcription.

### Catalase is epigenetically regulated in CLL cells

**Fig. 3:**
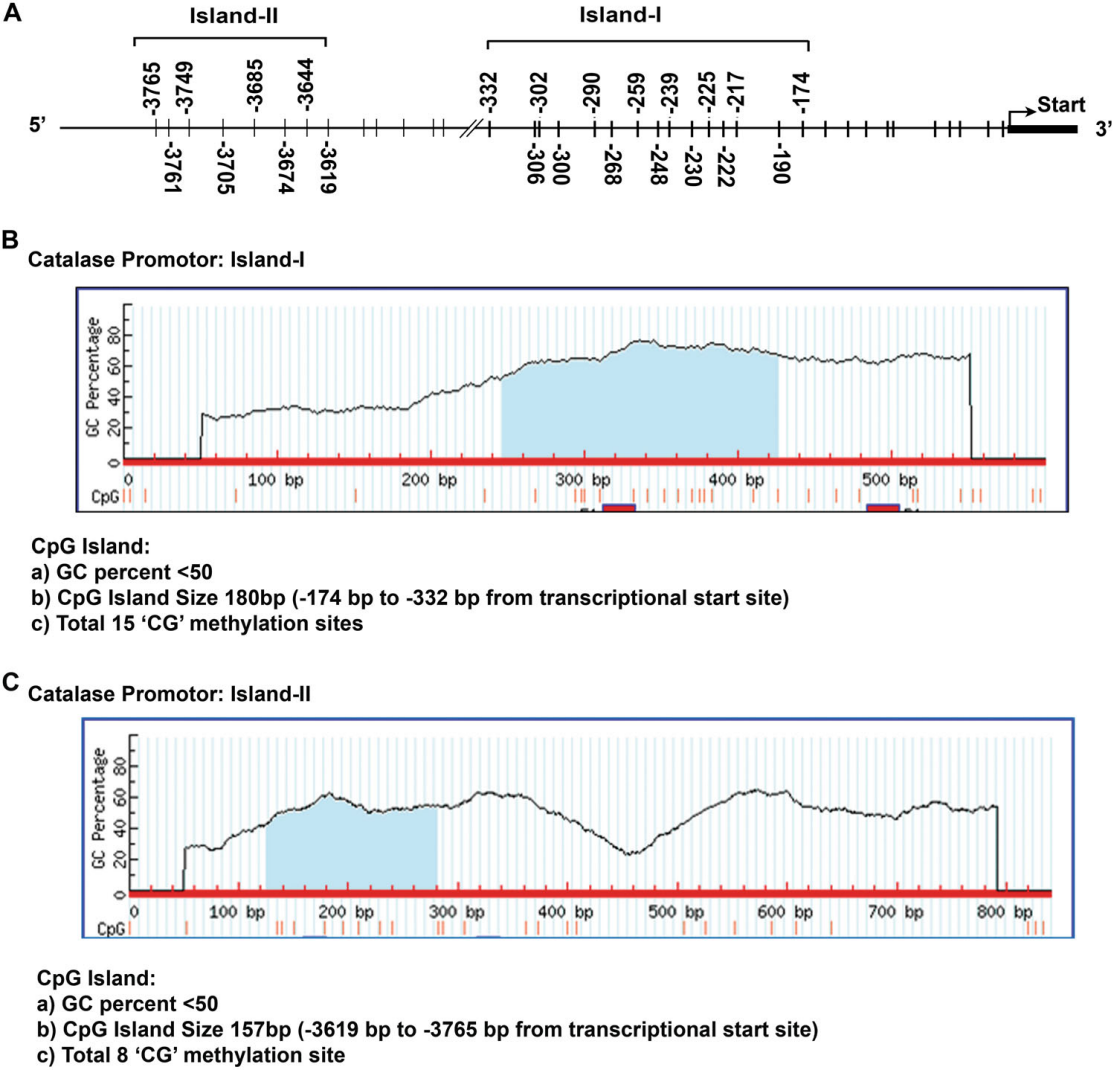
Diagram of catalase gene promoter and prediction of putative CpG-Islands. **A** The entire human catalase promoter was analyzed for the prediction of putative CpG sites for methylation (http://www.urogene.org/cgi-bin/methprimer/methprimer.cgi). Two potential CpG-Islands were detected in the catalase promoter: a proximal (Island-I; nt −174 to −332) and a distal (Island-II; nt −3619 to −3765) as shown by line drawing for putative methylation sites. **B**, **C** These diagrams are showing the GC contents, putative methylation sites, and positions in the human catalase promoter as per the analysis.

**Fig. 4:**
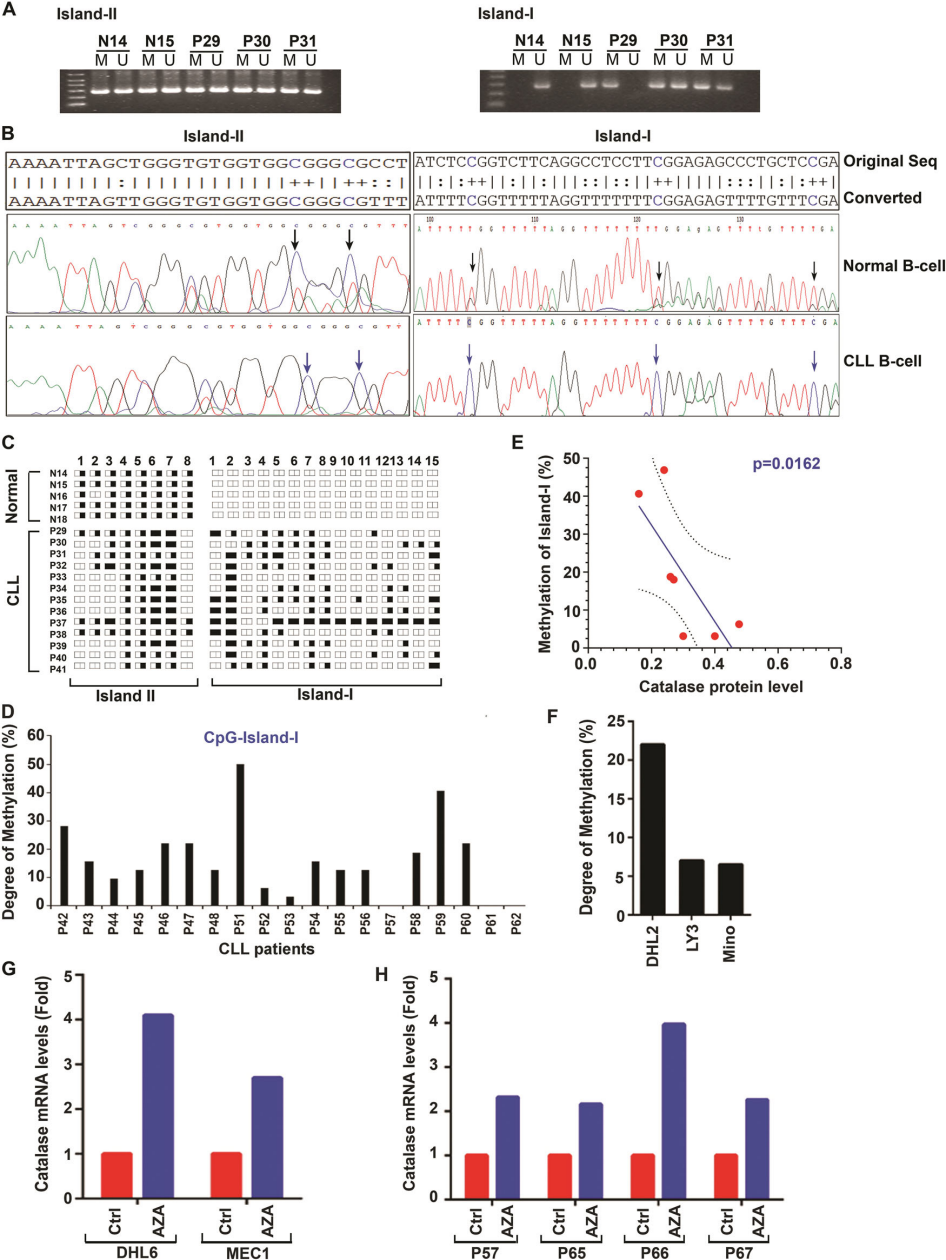
Catalase expression is epigenetically regulated in CLL cells. **A** Human catalase promoter possesses two distinct CpG-islands. Genomic DNA from normal B-cells (N14, N15) and CLL cells (P29–P31) were bisulfite converted and the predicted CpG-Islands in the catalase promoter were PCR amplified using methylation/unmethylation-specific (M/U) primer sets. PCR products were analyzed on agarose gels and photographed. **B**, **C** Methylation status in CpG-Island-I determines catalase expression in CLL cells. Genomic DNA from normal B-cells (*n* = 5; N14–N18) and CLL cells (*n* = 32; P29–P48, P51–P62) were bisulfite converted and the CpG-Island-I and -II in the catalase promoter were PCR amplified as described above and sequenced. Representative DNA sequence analyses from normal B-cells and CLL cells are shown and compared (**B**). A diagrammatic presentation of the methylation status in CpG-Island-I and –II of the catalase promoter in normal B-cells (*n* = 5; N14–N18) vs. CLL cells (*n* = 13; P29–P41) (obtained from the sequence analysis data) is shown in panel **C**. Each spliced rectangle (representing both alleles of the gene) indicates a putative CG-methylation site; half-filled rectangle represents methylation at one allele; filled rectangle represents methylation at both the alleles; empty rectangle indicates no methylation at either alleles. **D** CpG-Island-I in CLL cells is methylated differentially. Methylation status of the fifteen putative CpG sites in Island-I of the catalase promoter in CLL cells (*n* = 19; P42–P48, P51–P62) was determined by analyzing the sequence data described above and presented as “Degree of Methylation”. **E** Degree of Methylation in CpG-Island-I inversely correlates with catalase protein levels. CLL cell lysates as available from few CLL patients analyzed in panel D (P42–P45, P47, P48, P53) were examined for catalase expression in western blots. Normalized catalase protein levels (with respect to GAPDH) in CLL cells were further analyzed to find any correlation with the degree of methylation in CpG-Island-I of the catalase promoter. **F** Methylation status of CpG-Island-I in B-cell lymphoma cells. Similarly, degree of methylation in CpG-Island-I of the catalase promoter in other B-cell malignancies including diffuse large B-cell lymphoma (DHL2, LY3) and mantle cell lymphoma (Mino) was also determined. **G**, **H** Treatment of malignant B-cells with 5’-azacytidine increases catalase expression. DHL6/MEC1 cells (**G**) or primary CLL cells (**H**; *n* = 4; P57, P65–P67) were treated with a demethylating agent 5’-azacytidine (AZA; 1 µM) or left untreated. Catalase mRNA levels were determined by qRT-PCR from total RNA. GAPDH was used as internal control. Ct values were calculated and results are presented as “fold change” in treated vs. untreated cells.

To delineate the mechanism of reduced catalase mRNA levels in CLL cells, we first explored if catalase gene transcription was regulated epigenetically, for example, via methylation of the catalase promoter. Thus, we analyzed the entire catalase promoter for CpG-island prediction (http://www.urogene.org/cgi-bin/methprimer/methprimer.cgi)^23^. Two predicted “CpG-islands” were identified^32^: (i) Proximal (Island-I; nt −174 to −332) and (ii) Distal (Island-II; nt −3619 to −3765) containing 15 and 8 putative CpG methylation sites, respectively, in the catalase promoter (Fig. 3A–C). To find if these predicted methylation sites in the catalase promoter are methylated, genomic DNA from normal B-cells (*n* = 5) or CLL cells (*n* = 32) were bisulfite converted, PCR amplified and sequenced. Our results demonstrated methylation in the CpG-Island-II in both normal B-cells and CLL cells, while the CpG-Island-I of the catalase promoter exhibited variable degrees of methylation only in CLL cells, but not in normal B-cells (Fig. 4A–D). Further analysis finds a significant (*p* = 0.0162) inverse relationship (Spearman correlation coefficient is −0.85) between the degree of CpG methylation in Island-I of the catalase promoter and its protein levels in CLL cells (Fig. 4E). In addition, our results also suggested variable levels of methylation in the CpG-Island-I in DHL2, LY3, or Mino cells (Fig. 4F). In total, these findings suggest that methylation of the “CpG-Island-I” may regulate catalase expression in CLL cells.

Finally, to interrogate if the catalase promoter methylation plays a functional role in regulating its transcription, cells were treated in vitro with 5’-azacytidine (AZA) either alone (MEC1 and DHL6) or together with IL-2/IL-15/CpG ODN (CLL cells; *n* = 4)^24^, and catalase mRNA level was determined by qRT-PCR. Indeed, we detected increase of catalase transcript levels in both MEC1/DHL6 cells (Fig. 4G) as well as in primary CLL cells (Fig. 4H) treated with AZA. Thus, findings from these series of experiments suggest that catalase expression in CLL cells is regulated epigenetically, at least in part, via promoter methylation specifically of the “CpG-Island-I”.

### Enforced induction of ROS activates AXL signaling axis

**Fig. 5:**
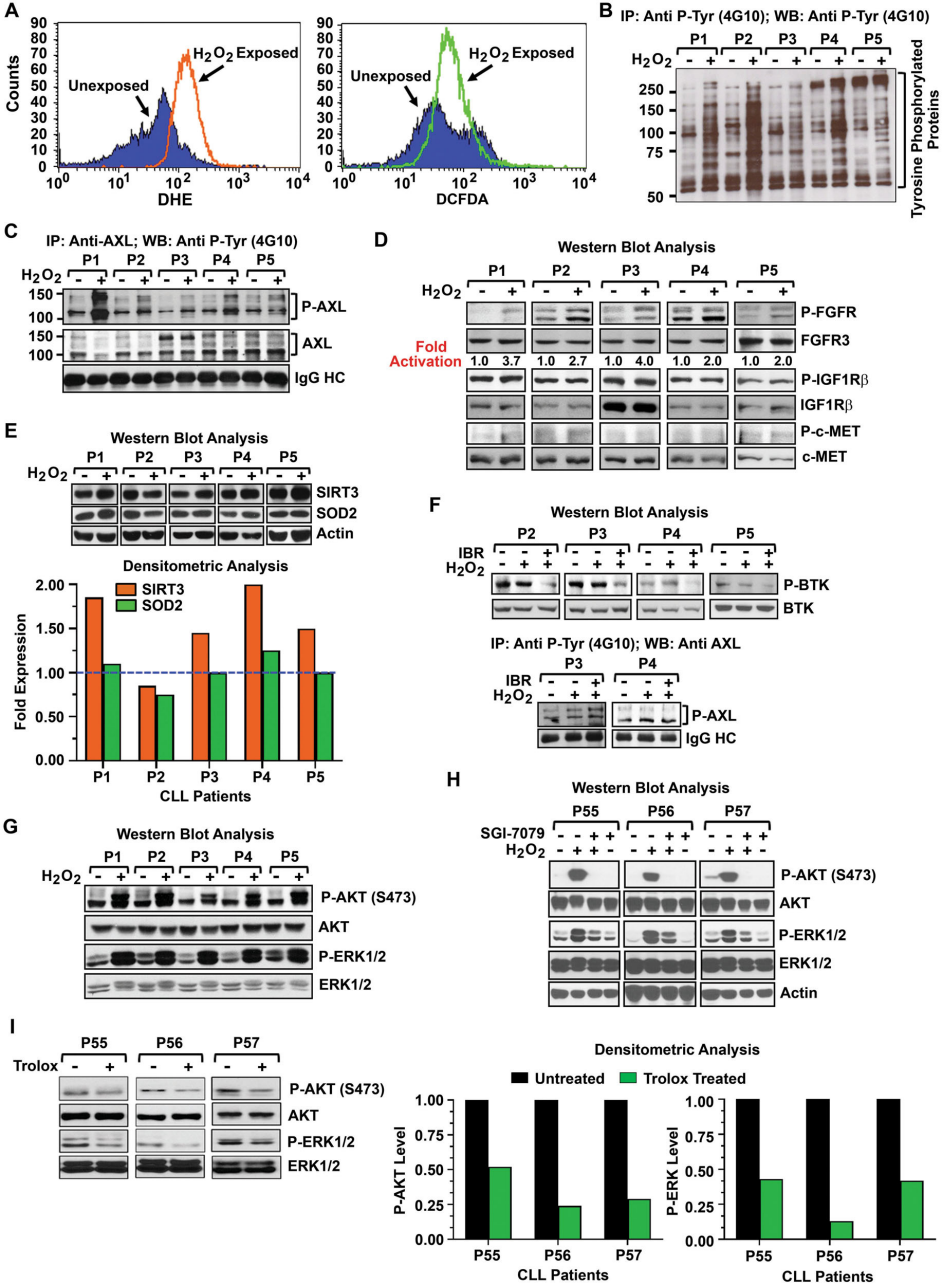
Induction of ROS activates AXL signaling axis in CLL cells. **A** In vitro generation of ROS in CLL cells. Purified CLL cells were exposed to H_2_O_2_ (0.6 mM) for 5 min or left untreated. Cells were analyzed for ROS generation by flow cytometry after staining the cells with DHE to detect O_2_^−^ generation or DCFDA to detect H_2_O_2_ accumulation. Results of ROS accumulation in CLL cells from a representative CLL patient is shown. **B** ROS generation increases tyrosine phosphorylation levels. Purified CLL cells (*n* = 5; P1–P5) treated with H_2_O_2_ as described above were lysed and tyrosine-phosphorylated proteins were immunoprecipitated from the cell lysates using a phospho-tyrosine (4G10) antibody, followed by western blot analysis using the same antibody. **C** Enforced induction of ROS activates AXL. CLL cell lysates (P1–P5) used in panel **B** were analyzed to detect AXL phosphorylation levels by immunoprecipitating AXL, followed by western blot using 4G10. The blot was stripped and reprobed with an antibody to AXL. IgG heavy chain (HC) was used as loading control. **D** Impact of ROS accumulation on other RTKs. CLL cell lysates (P1–P5) used in panels **B**, **C** were further analyzed to detect the activation status of multiple RTKs including FGFR (a downstream target of AXL), IGF1Rβ and c-MET in western blots using phospho-specific antibodies. Respective blots were stripped and probed with a specific antibody to FGFR3, IGF1Rβ, or c-MET, and used as loading controls. Densitometric analysis was performed to detect “fold activation” of FGFR (P-FGFR: FGFR3). **E** Status of SIRT3 and SOD2 expression in H_2_O_2_-exposed CLL cells. The above CLL cell lysates were further analyzed for the expression of SIRT3 and SOD2 in western blots using specific antibodies. Actin was used as loading control. Densitometric analyses were performed to determine the expression levels of SIRT3 (SIRT3: actin) and SOD2 (SOD2: actin), and presented as “fold-expression” relative to the basal level the value of which was arbitrarily taken as “1”. The dotted line indicates basal levels (bottom panel). **F** Enforced induction of ROS does not activate BCR signal. Purified CLL cells used above (P2–P5) were treated with ibrutinib (0.75 µM) for 1 h prior exposing the cells to H_2_O_2_ as described above for 5 min or left untreated/unexposed. Cell lysates were analyzed for the activation of BTK (as an indicator for BCR signal activation) in western blots. The blots were stripped, probed for BTK and used as loading control. Further, phospho-tyrosine proteins were immunoprecipitated from the above cell lysates (P3, P4), followed by western blot analysis to detect activation of AXL using a specific antibody. IgG HC was used as loading control (bottom panel). **G** Impact of ROS-mediated AXL activation on its downstream signal mediators. Finally, H_2_O_2_-treated CLL cell lysates (P1–P5) used above (**B**–**E**) were assessed for the activation status of AXL downstream targets, AKT and ERK1/2, in western blots using phospho-specific antibodies. The blots were stripped and reprobed to detect total AKT or ERK1/2. **H** Targeting AXL inhibits ROS-induced activation of AKT/ERK1/2. Purified CLL cells (P55–P57) pretreated with an AXL-inhibitor SGI-7079 at a sublethal dose or left untreated were exposed to H_2_O_2_ for 5 min. Cell lysates were examined for the activation status of AKT and ERK1/2 in western blots using specific antibodies. The blots were stripped and reprobed to detect total AKT or ERK1/2. Actin was also used as loading control. **I** Inhibition of endogenous ROS reduces phosphorylation levels of AKT/ERK1/2. Purified CLL cells (P55–P57) were treated with a ROS-inhibitor, Trolox for 4 h. Cell lysates were analyzed for the activation status of AXL downstream signal mediators, AKT and ERK1/2, in western blots using specific antibodies. The blots were stripped and reprobed to detect total AKT or ERK1/2. Densitometric analyses were performed to assess modulation of AKT (P-AKT: AKT) or ERK1/2 (P-ERK1/2: ERK1/2) activation in Trolox treated vs. untreated CLL cells and presented as bar diagrams (right panels). For each sample, the basal level activation was arbitrarily taken as “1”.

Accumulation of ROS has been shown to activate several RTKs in a ligand-independent manner resulting in cell growth^33–35^. It is now well established that H_2_O_2_ acts as a second messenger for signal transduction and signal amplification. Thus, we interrogated if increased production of H_2_O_2_ could activate the AXL signaling axis, which is constitutively active and critical for CLL cell survival^21^. For this, CLL cells were treated with H_2_O_2_ for 5 min and generation of ROS was determined by flow cytometry. Results showed a subtle increase of ROS in H_2_O_2_-treated vs. untreated CLL cells from basal levels (Fig. 5A). Next, we determined if enforced increase of ROS had any impact on tyrosine-phosphorylated proteins in H_2_O_2_-treated vs. untreated CLL cells. Indeed, we detected substantial increase of tyrosine phosphorylation levels on several proteins in H_2_O_2_-treated CLL cells (Fig. 5B) including AXL (Fig. 5C) and FGFR (Fig. 5D), but not on Tyro3 (data not shown) or other RTKs like c-MET or IGF1Rβ (Fig. 5D). Given our finding that AXL positively regulates FGFR signal^22^, increase of FGFR phosphorylation levels in H_2_O_2_-treated CLL cells (Fig. 5D) was likely as a result of AXL activation by ROS (Fig. 5C). Other tyrosine-phosphorylated protein bands (<100 kDa) appeared on the blot (Fig. 5B) may be the downstream targets of AXL including LYN (56 kDa), SYK (72 kDa), and/or ZAP70 ^20^.

As ROS can also activate SIRT3 expression^15,29^ and our findings that long-term exposure (4 h) of CLL cells to H_2_O_2_ may result in SIRT3 upregulation (Fig. 1F), we further analyzed the H_2_O_2_-exposed CLL cell lysates used above (P1–P5) to assess the expression status of SIRT3 and SOD2 in western blots. A low to moderate increase (~1.5–2-fold) of SIRT3 expression was discernible in CLL cells (4 of 5) within a 5-min of exposure to H_2_O_2_, while no significant alteration of SOD2 expression was detected (Fig. 5E). Whether this elevation of SIRT3 expression in H_2_O_2_-exposed CLL cells is linked to AXL activation remains to be elucidated.

Given that BCR pathway is critical for CLL cell survival/proliferation and that, several of its downstream signaling components are the targets for therapy^36^, we investigated the impact of enforced ROS accumulation on BCR signal. Thus, purified CLL cells used in panels B–D (P2–P5) were pretreated with ibrutinib for 1 h prior exposing the cells to H_2_O_2_ for 5 min or left untreated/unexposed. Cell lysates were analyzed to detect phosphorylation status of BTK (as an indicator of BCR activation) in western blot. While H_2_O_2_-treatment could not activate BTK from its basal level (upper panel, Fig. 5F), it activated AXL (lower panel, Fig. 5F), consistent with the findings in Fig. 5C, which remained unaffected prior ibrutinib treatment. In total, these results suggest that accumulation of ROS may activate AXL but not the BCR signal in CLL cells and that, ibrutinib treatment may not have any impact on ROS-induced AXL activation.

Further analysis of the H_2_O_2_-exposed CLL cell lysates used in panels B–E demonstrated a robust activation of AKT and ERK1/2, downstream signal mediators of AXL (Fig. 5G). Indeed, H_2_O_2_-mediated activation of AKT/ERK in CLL cells was completely inhibited upon pretreatment of the cells with a high-affinity AXL inhibitor^25^ (Fig. 5H). As most kinase inhibitors show off-target effects, we pretreated purified CLL cells (*n* = 2) with an AXL inhibitor^21^, a BCR-ABL inhibitor asciminib or a JAK2 inhibitor fedratinib for 2 h prior exposing to H_2_O_2_. Cell lysates were analyzed for P-AKT and P-ERK1/2 in western blots. Interestingly, we detected a patient-specific effects of the inhibitors on AKT/ERK activation (Supplementary Fig. S2). While pretreatment of CLL cells with an AXL inhibitor reduced H_2_O_2_-induced AKT/ERK activation levels in both the samples, asciminib completely inhibited P-AKT level in CLL cells from one patient but not in other (Supplementary Fig. S2). Fedratinib pretreatment, on the other hand, did not show any inhibitory effect on H_2_O_2_-induced AKT activation in CLL cells obtained from both the patients. Further analysis suggests that asciminib and fedratinib are able to target ERK1/2 activation (Supplementary Fig. S2). A recent in vivo study demonstrated that cardiotoxicity induced by Ponatinib, another BCR-ABL inhibitor, was a result of direct inhibition of AKT/ERK signaling axes by the targeted-agent in cardiomyocytes^37^. Although this study suggested asiminib as a potentially much less cardiotoxic, we believe that asciminib may exert patient-specific inhibitory effects on AKT/ERK in CLL cells. However, treatment of CLL cells with a ROS-inhibitor inhibited both P-AKT and P-ERK1/2 (Fig. 5I). Taken together, these results suggest that CLL cells may escape the detrimental cytotoxic effects of increased ROS accumulation by activating key cell survival signaling pathways including AXL.

## Discussion

Compared with normal cells, cancer cells have higher levels of ROS, which seem to be required for malignant initiation and progression^38^, and the unchecked ROS accumulation is thought to play a part in the conversion from normal hematopoietic stem cells to leukemic cells^39,40^. The major endogenous source of cellular ROS is the mitochondrial electron transport chain, where continuous electron leakage to O_2_ occurs during aerobic respiration, generating O_2_^−^^41^. Although mitochondrial activities are higher in CLL cells^19^, we have detected significantly lower levels of O_2_^−^ in CLL cells as compared to normal B-cells, suggesting a rapid conversion of O_2_^−^ into H_2_O_2_ by the mitochondrial superoxide detoxification enzyme SOD2, which likely remains as a highly active enzyme in CLL cells.

While the known primary regulation of SOD2 is through transcriptional activation, Chen et al.^15^ demonstrated that acetylation of SOD2 at K68 residue significantly reduces its activity while SIRT3 binds to, deacetylates and activates SOD2. In consistent with this report, we detected a marked reduction of acetylated-SOD2 levels in CLL cells indicating its constitutive enzymatic activity and that, the class-III histone deacetylase SIRT3 remained overexpressed, although at variable levels. While the precise mechanism of SIRT3 overexpression in CLL cells remains to be elucidated, we found that BCR stimulation could induce rapid upregulation of SIRT3 in CLL cells. However, we believe that increased ROS accumulation may also activate and maintain constitutive levels of SIRT3 in CLL cells as indicated in earlier studies^15,29^. Whether ROS-induced SIRT3 upregulation in CLL cells is linked to AXL activation remains to be elucidated.

Despite significant reduction of O_2_^−^ levels, we found increased accumulation of H_2_O_2_ in CLL cells. To delineate the mechanism of aberrant H_2_O_2_ levels in CLL cells, we examined the expression status of catalase, which protects the cells by removing H_2_O_2_. Our analysis finds reduced levels of mRNA and protein of catalase in CLL cells from majority of patients tested, suggesting that reduced expression of catalase may be responsible for H_2_O_2_ accumulation in these leukemic cells. In an effort to decipher the mechanism of catalase downregulation, we identified two putative CpG-islands upon analysis of its promoter. Our results indicated that the CpG-Island-I may be critical to regulate catalase transcription as variable degrees of allele-specific methylation in this region were detectable only in CLL cells but, not in normal B-cells. In addition, treatment of primary CLL cells or MEC1 cells with AZA increased catalase mRNA levels, further suggesting that promoter methylation regulates catalase level in CLL cells, at least in part. It is also noteworthy that SIRT3 overexpression has been shown to induce expression of antioxidant genes like SOD2 or catalase via activation of FOXO3a^16^. However, we did not find any positive association of SIRT3 overexpression with catalase or SOD2 levels in CLL cells obtained from previously untreated CLL patients (*n* = 10) (Supplementary Fig. S3); suggesting that SIRT3 mediated regulation of SOD2 or catalase gene transcription may be context-dependent and/or cell-type specific.

Oxidative stress, even at low levels, can activate RTKs including receptors for epidermal growth factor (EGF), platelet-derived growth factor (PDGF) and FGF in a ligand-independent manner resulting in cell growth^33–35^. Interestingly, treatment of vascular smooth muscle cells with exogenous H_2_O_2_ induced activation of AXL, independent of its ligand Gas6^26,42^. Thus, elevation of ROS in CLL cells certainly provides them with some advantages over normal B-cells. Indeed, we detected that enforced elevation of ROS in CLL cells activated AXL and its downstream targets FGFR^22^, AKT and ERK1/2, but not IGF1Rβ or c-MET. Earlier studies also demonstrated that H_2_O_2_ activated EGFR^34^, PDGFR^33^, and FGFR^43^ at concentrations ranging from 5–20 mM in vitro^42^. In contrast, we detected a robust activation of AXL upon treatment of CLL cells with H_2_O_2_ at 8–30-fold lower dose suggesting that AXL may be highly sensitive to ROS elevation and that, activation of FGFR in CLL cells in response to H_2_O_2_ is likely to be a consequence of AXL activation; not a direct effect of H_2_O_2_ on FGFR. Protein tyrosine phosphatases, which are responsible for dephosphorylation RTKs, can be inactivated by ROS-mediated oxidation, leading to elevated phosphorylation of the target proteins^5^. However, only a subtle reduction of total phosphatase levels was discernible in CLL cells in response to in vitro H_2_O_2_ treatment (Supplementary Fig. S4). Further study is needed to elucidate the mechanism of targeted activation of AXL in CLL cells in response to increased ROS.

In summary, we detected elevated levels of H_2_O_2_ but significantly lower levels of O_2_^−^ in CLL cells, which could be attributable to reduced catalase levels and overexpression of SIRT3, respectively. In contrast to the superoxide anion and hydroxyl radical, the less reactive H_2_O_2_ is not only involved in many physiological processes, such as hypoxic signal transduction, cell differentiation, and proliferation but also plays a role in mediating immune responses. However, it exerts its effects depending on the cellular context, its local concentration as well as its exposure time. Thus H_2_O_2_ is no more considered as an unwanted rather toxic byproduct, but plays an important role in the control of vital cellular processes. Thus, we believe that generation of high level of superoxide anion may be detrimental to the cells while low level accumulation of H_2_O_2_ may play a vital role to prolong cancer cells’ survival in toxic environment. Given our findings, we also believe that CLL cells have adopted a process to rapidly neutralize superoxide anion accumulation (via SIRT3/SOD2) but utilize H_2_O_2_ to prolong their survival by activating cell survival pathways.

Efficient removal of O_2_^−^ from the cellular environment is usually a beneficial outcome; increased accumulation of H_2_O_2_ due to reduced catalase expression while detrimental to normal cells but may promote genetic instability and cell signaling in malignant cells including CLL cells. It is, however, tempting to speculate about the formation of a ROS gradient from the source of production, where proteins under the influence of such a gradient would be oxidized, while others out of reach would be immune to increases in the level of ROS^44^. Of relevance, prolonged production of ROS in response to BCR stimulation has been shown to promote B-cell activation and proliferation^45^ and that, activated cells also generate ROS as a byproduct of normal mitochondrial respiration^46^. In addition, activation of the PI3K/mTOR pathway by BCR-ABL contributes to increased production of mitochondrial ROS in BCR-ABL-transfected murine pre-B-cells and human megakaryocytes^47^. In agreement with these studies and given our findings, we speculate that constitutive activation of the BCR signal in CLL cells may upregulate SIRT3 and maintain its levels by generating ROS which, on the other hand, promotes cell survival through activation of RTKs like AXL.

**Fig. 6:**
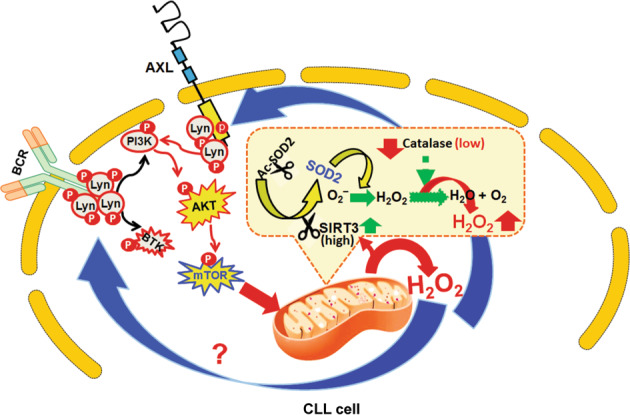
Proposed model of ROS generation and regulation of survival signals in CLL cells. Data presented in this study demonstrate that CLL cells contain elevated levels of H_2_O_2_ with significantly reduced O_2_^−^ levels compared to normal B-cells. This is primarily due to the presence of highly active mitochondrial SOD2 enzyme in CLL cells converting O_2_^−^ into H_2_O_2_ more rapidly. Overexpression of SIRT3 in CLL cells deacetylates SOD2 and likely maintains its constitutive enzymatic activity. Moreover, we have also detected reduced expression of catalase in CLL cells causing H_2_O_2_-accumulation. Although we have identified two distinct CpG-Islands (I and II) for methylation in the human catalase promoter, our results suggest that it is the Island-I, which primarily regulates catalase levels in CLL cells. Indeed, we have detected variable degrees of methylation in CpG-Island-I of the catalase promoter only in CLL cells and not in normal B-cells and that, catalase expression is reduced in CLL cells both at mRNA and protein levels. While released from mitochondria, H_2_O_2_ may activate the AXL/FGFR/AKT/ERK signaling axis and/or the BCR pathway, independent of ligand, and maintain constitutive activation levels of these cell survival pathways through a positive feedforward loop. For example, activation of AKT/mTOR pathway leads to increase of oxygen consumption and mitochondrial metabolic activities and thus, generation of ROS. Of note, we found that ROS generation or BCR activation induces SIRT3 expression in CLL cells. Therefore, one potential therapeutic intervention can be to increase ROS-scavenging capacity using antioxidants, thereby abrogating ROS signaling and suppressing tumor growth in combination with current CLL therapies.

In conclusion, we propose that constitutively active known cell survival signaling pathways (BCR/AXL) in CLL cells may activate and maintain elevated levels of ROS via PI3K/AKT/mTOR axis (Fig. 6), a key regulator of mitochondrial oxygen consumption and oxidative capacity^48^. On the other hand, to escape from the detrimental effect of overproduced O_2_^−^, CLL cells adopted a mechanism to eliminate O_2_^−^ more efficiently by converting inactive SOD2 into a constitutively active enzyme via inducing SIRT3 expression, while maintaining low levels of catalase through variable degrees of promoter methylation for H_2_O_2_ accumulation to promote cell survival (Fig. 6). Thus, increase of ROS in CLL cells may not only activate AXL signaling axis but also upregulate SIRT3 to maintain this positive feedback loop. Therefore, one potential therapeutic intervention can be to increase ROS-scavenging capacity using antioxidants, thereby abrogating ROS signaling and suppressing tumor growth in combination with current CLL therapies. However, this hypothesis warrants further investigation.

## Supplementary information

Checklist

Supplementary Methods

Supplementary Figure Legends

Supplementary Table S1

Supplementary Table S2

Supplementary Figure S1

Supplementary Figure S2

Supplementary Figure S3

Supplementary Figure S4
