# Supplementary material for: SIRT3 overexpression and epigenetic silencing of catalase regulate ROS accumulation in CLL cells activating AXL signaling axis
Source: Blood Cancer J. 2021 May 17;11(5):93. doi: 10.1038/s41408-021-00484-6 (PMC8129117; doi:10.1038/s41408-021-00484-6)
Supplement: Supplementary file 2 — Supplementary Methods [file 41408_2021_484_MOESM2_ESM.docx]

**SUPPLEMENTARY METHODS**

**Phosphatase assay**

Purified CLL cells from previously untreated CLL patients (P55 – P59) were treated with 0.6mM H_2_O_2_ for 5 min and cell lysates were prepared. Protein content was measured by BCA method. Total phosphatase (alkaline phosphatase, acid phosphatase, tyrosine phosphatase or serine-threonine phosphatase) activity was measured in CLL cell lysates using a phosphatase assay kit (G-Biosciences) according to manufacturer’s instructions.

**Isolation of mitochondrial fraction and western blot**

Please see the “Materials and Methods” section in the main text.

**Treatment of CLL cells with Kinase inhibitors and H_2_O_2_**

Purified CLL cells from previously untreated CLL patients (P70, P71) were pre-treated with a high-affinity AXL inhibitor (TP-0903), a BCR-ABL inhibitor asciminib or a JAK2 inhibitor fedratinib for 2 hours prior exposing to H_2_O_2_ (0.6mM) for 5 minutes. Cell lysates were analyzed for P-AKT and P-ERK1/2 in western blots.
