# Supplementary material for: SIRT3 overexpression and epigenetic silencing of catalase regulate ROS accumulation in CLL cells activating AXL signaling axis
Source: Blood Cancer J. 2021 May 17;11(5):93. doi: 10.1038/s41408-021-00484-6 (PMC8129117; doi:10.1038/s41408-021-00484-6)
Supplement: Supplementary file 3 — Supplementary Figure Legends [file 41408_2021_484_MOESM3_ESM.docx]

**SUPPLEMENTARY FIGURE LEGENDS**

**Supplementary Figure S1. Purity assessment of mitochondrial fraction preparations from normal B-cells and CLL cells.** Mitochondrial and cytoplasmic fractions isolated from purified normal B-cells (N1 – N4) or CLL cells (P6, P10, P11) as described in the “Methods” section and used in Figure 1C were analyzed to detect the mitochondrial-specific protein VDAC in western blots using a specific antibody. Results suggest that the mitochondrial fractions isolated from the cells were highly purified based on the detection of VDAC only in mitochondrial fractions and not in cytoplasmic fractions. Equal amount of protein from each sample was loaded based on the BCA protein estimation method.

**Supplementary Figure S2. Impact of tyrosine kinase inhibitors on H_2_O_2_-induced activation of AKT and ERK1/2.** Purified CLL cells from previously untreated CLL patients (P70, P71) pre-treated with the individual kinase inhibitors as indicated (described in the “supplementary methods”) were exposed to H_2_O_2_ for 5 minutes. Cell lysates were analyzed for the activation status of AKT and ERK1/2 in western blots using specific antibodies. Total AKT and ERK1/2 were used as loading controls.

**Supplementary Figure S3. Assessment of relative expression levels of SIRT3, SOD2 and catalase in CLL cells.** Lysates of purified CLL cells from previously untreated CLL patients (n=10; P69, P72 – P80) were analyzed for the expression of catalase, SOD2 and SIRT3 in western blots using specific antibodies. Acetylation status of SOD2 at K68 residue was also assessed using a specific antibody to Ac-SOD2 (K68). GAPDH was used as loading control. Densitometric analyses were performed to determine expression levels of catalase, SOD2, SIRT3 or Ac-SOD2 with respect to GAPDH or total SOD2 (for Ac-SOD2), respectively (right panels). Available prognostic factors of the CLL patients are shown for comparison (left bottom panel).

**Supplementary Figure S4. Impact of H_2_O_2_-treatment on phosphatase activity in CLL cells.** Freshly isolated, purified CLL cells from previously untreated CLL patients (P55 – P59) were treated with 0.6mM H_2_O_2_ for 5 min or left untreated and cell lysates were analyzed for the total phosphatase activity using a phosphatase assay kit (G-Biosciences) according to the manufacturer’s protocol. Total phosphatase activity is presented as nmole/min/mg of protein.
