# Supplementary material for: SIRT3 overexpression and epigenetic silencing of catalase regulate ROS accumulation in CLL cells activating AXL signaling axis
Source: Blood Cancer J. 2021 May 17;11(5):93. doi: 10.1038/s41408-021-00484-6 (PMC8129117; doi:10.1038/s41408-021-00484-6)
Supplement: Supplementary file 4 — Supplementary Table S1 [file 41408_2021_484_MOESM4_ESM.docx]

**Supplementary Table S1. Characteristics of the CLL patients used in this study**

| **CLL Pt.** | **Sex** | **Age** | **Rai Stage** | **IGVH Mutation Status** | **ZAP70** | **CD38** | **FISH** |
| --- | --- | --- | --- | --- | --- | --- | --- |
| P1 | F | 67 | 0 | Unmutated | Negative | Negative | 13q- |
| P2 | M | 78 | IV | Mutated | Negative | Positive | Normal |
| P3 | F | 63 | IV | Not Done | Positive | Negative | 17p- |
| P4 | F | 91 | 0 | Mutated | Negative | Negative | 13q- |
| P5 | M | 59 | 0 | Mutated | Negative | Negative | Normal |
| P6 | F | 59 | III | Unmutated | Positive | Negative | Normal |
| P7 | M | 65 | IV | Mutated | Negative | Negative | 13q- |
| P8 | M | 75 | I | Unmutated | Negative | Negative | 13q- |
| P9 | F | 74 | 0 | Mutated | Negative | Negative | Normal |
| P10 | M | 73 | 0 | Mutated | Negative | Negative | 17p- |
| P11 | F | 67 | II | Unmutated | Negative | Negative | 13q- |
| P12 | M | 55 | 0 | Mutated | Negative | Negative | 13q- |
| P13 | M | 76 | 0 | Mutated | Negative | Negative | 13q- |
| P14 | M | 55 | 0 | Mutated | Negative | Negative | 13q- |
| P15 | M | 59 | I | Unmutated | Positive | Positive | 11q- |
| P16 | M | 77 | III | Mutated | Positive | Negative | 17p- |
| P17 | F | 69 | 0 | Mutated | Negative | Negative | Trisomy 12 |
| P18 | F | 72 | II | Mutated | Negative | Positive | Normal |
| P19 | M | 79 | 0 | Mutated | Negative | Negative | 13q- |
| P20 | M | 79 | IV | Mutated | Negative | Positive | Normal |
| P21 | M | 57 | 0 | Mutated | Negative | Negative | Normal |
| P22 | M | 67 | 0 | Mutated | Negative | Negative | 13q- |
| P23 | M | 75 | I | Unmutated | Negative | Negative | 13q- |
| P24 | F | 48 | I | Unmutated | Positive | Negative | Normal |
| P25 | F | 52 | 0 | Unmutated | Positive | Negative | 13q- |
| P26 | M | 57 | 0 | Unmutated | Positive | Positive | Normal |
| P27 | M | 64 | 0 | Unmutated | Positive | Negative | 17p- |
| P28 | M | 66 | I | Unmutated | Positive | Negative | Normal |
| P29 | M | 71 | I | Unmutated | Positive | Negative | 11q- |
| P30 | F | 85 | 0 | Mutated | Negative | Negative | 17p- |
| P31 | M | 64 | I | Unmutated | Positive | Negative | Trisomy 12 |
| P32 | M | 71 | III | Mutated | Negative | Negative | 13q- |
| P33 | F | 58 | 0 | Mutated | Negative | Negative | 13q- |
| P34 | M | 69 | 0 | Mutated | Negative | Negative | 13q- |
| P35 | M | 63 | 0 | Mutated | Negative | Negative | 13q- |
| P36 | M | 66 | 0 | Uninterpret | Negative | Negative | Normal |
| P37 | M | 65 | IV | Mutated | Negative | Negative | 13q- |
| P38 | M | 57 | 0 | Mutated | Negative | Negative | Normal |
| P39 | F | 91 | 0 | Mutated | Negative | Negative | 13q- |
| P40 | M | 68 | IV | Unmutated | Positive | Positive | 13q- |
| P41 | M | 70 | II | Unmutated | Positive | Positive | 13q- |
| P42 | M | 67 | I | Unmutated | Negative | Negative | 11q- |
| P43 | M | 73 | 0 | Mutated | Negative | Negative | 13q- |
| P44 | M | 72 | 0 | Mutated | Negative | Positive | Trisomy 12 |
| P45 | F | 66 | 0 | Not Done | Negative | Negative | 13q- |
| P46 | M | 79 | IV | Mutated | Negative | Negative | 13q- |
| P47 | M | 78 | 0 | Not Done | Not Done | Negative | Normal |
| P48 | M | 66 | I | Not Done | Positive | Positive | Not Done |
| P49 | F | 80 | I | Not Done | Not Done | Negative | t(11;14) |
| P52 | M | 56 | IV | Not Done | Not Done | Positive | 17p- |
| P53 | M | 86 | 0 | Not Done | Not Done | Negative | Normal |
| P54 | F | 44 | 0 | Mutated | Not Done | Positive | Trisomy 12 |
| P55 | M | 72 | 0 | Not Done | Negative | Negative | 13q- |
| P56 | M | 69 | I | Not Done | Not Done | Negative | Not done |
| P57 | M | 49 | IV | Unmutated | Positive | Negative | 17p- |
| P58 | M | 74 | I | Not Done | Not Done | Positive | Trisomy 12 |
| P59 | M | 88 | 0 | Not Done | Not Done | Not Done | 13q- |
| P60 | M | 69 | 0 | Mutated | Not Done | Not Done | 17p- |
| P61 | M | 63 | 0 | Not Done | Not Done | Positive | Not done |
| P62 | F | 63 | 0 | Not Done | Negative | Not Done | 13q- |
| P63 | M | 65 | I | Not Done | Not Done | Positive | 13q- |
| P64 | M | 71 | Not Done | Not Done | Not Done | Not Done | Trisomy 12 |
| P65 | F | 63 | Not Done | Not Done | Not Done | Positive | 13q- |
| P66 | M | 68 | IV | Not Done | Not Done | Not Done | Not done |
| P67 | M | 58 | III | Not Done | Not Done | Not Done | 13q- |
| P68 | M | 51 | 0 | Not Done | Not Done | Positive | Not done |
| P69 | M | 71 | II | Not Done | Not Done | Not Done | Not done |

**Note:** CD38 positivity: >= 20%; ZAP70 positivity: >= 20%
