# Supplementary material for: SIRT3 overexpression and epigenetic silencing of catalase regulate ROS accumulation in CLL cells activating AXL signaling axis
Source: Blood Cancer J. 2021 May 17;11(5):93. doi: 10.1038/s41408-021-00484-6 (PMC8129117; doi:10.1038/s41408-021-00484-6)
Supplement: Supplementary file 5 — Supplementary Table S2 [file 41408_2021_484_MOESM5_ESM.docx]

**Supplementary Table S2.** Primers used to sequence CpG-Islands in the human catalase promoter

| **Purpose** | **Name** | **Sequence** | **Region** |
| --- | --- | --- | --- |
| MSP sequencing | Cat 250 M FP | 5’-CGTTTGTAAAATTGGTAGGTTATTAACGGTC-3’ | Island-I |
| MSP sequencing | Cat 250 U FP | 5’-TGTTTGTAAAATTGGTAGGTTATTAATGGTT-3’ | Island-I |
| MSP sequencing | Cat 250 M/U RP | 5’-AAACAAACCAAAATTAAAAACCCAATAAAC-3’ | Island-I |
| MSP sequencing | Cat 3.5K M FP | 5’-CGTTTGTAATTTTAGTATTTTGGGAGGTC-3’ | Island-II |
| MSP sequencing | Cat 3.5K U FP | 5’-TGTTTGTAATTTTAGTATTTTGGGAGGTT-3’ | Island-II |
| MSP sequencing | Cat 3.5K M/U RP | 5'-CTCTATCACCCAAACTAAAATACAAC-3' | Island-II |

**Note:** Cat: catalase; MSP: methylation specific PCR; M: Methylation specific primer; U: Unmethylation specific primer; FP: Forward primer; RP: reverse primer.
