# Supplementary figures and images for: SIRT3 overexpression and epigenetic silencing of catalase regulate ROS accumulation in CLL cells activating AXL signaling axis
Source: Blood Cancer J. 2021 May 17;11(5):93. doi: 10.1038/s41408-021-00484-6 (PMC8129117; doi:10.1038/s41408-021-00484-6)

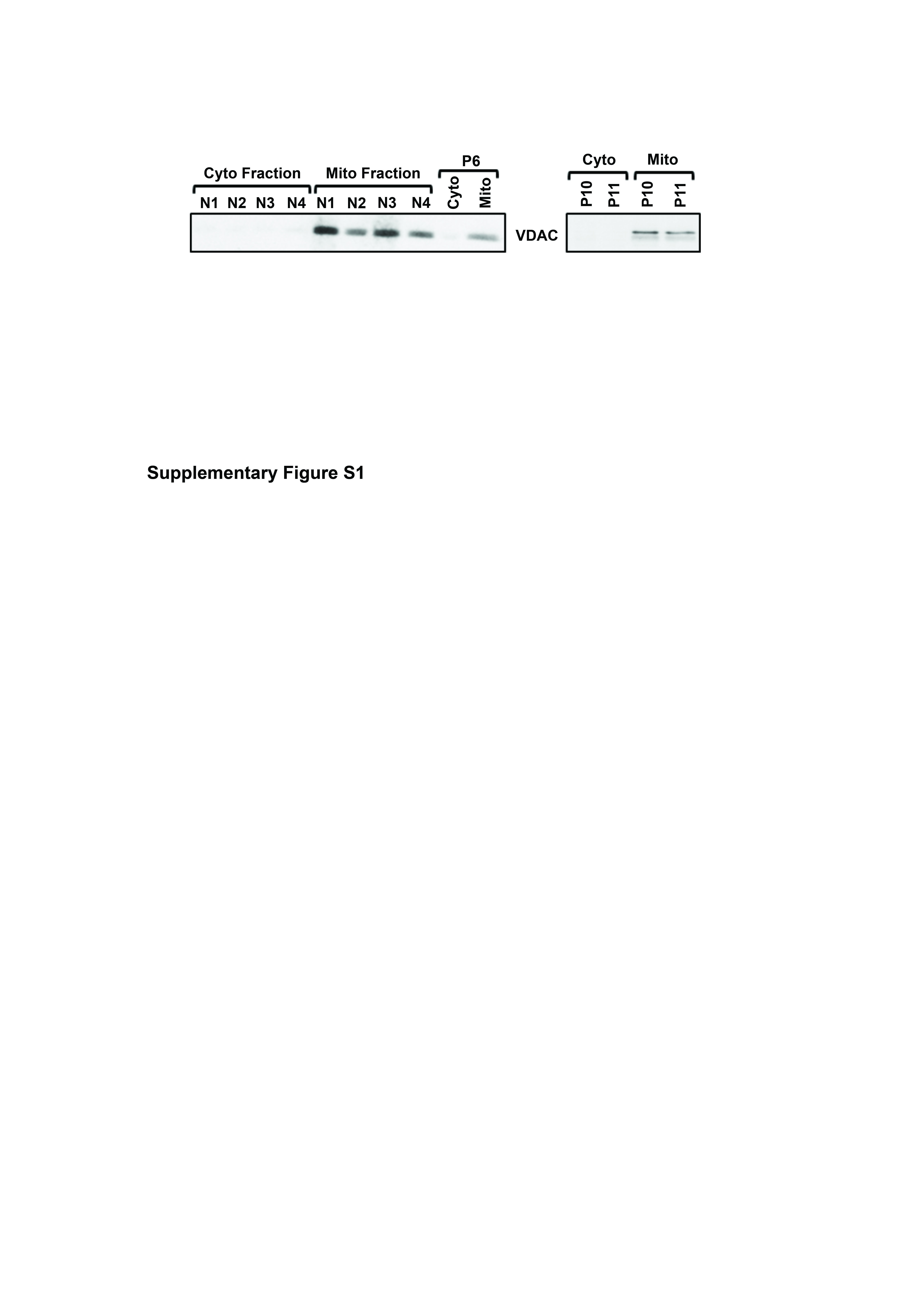

Supplement: Supplementary file 6 — Supplementary Figure S1 [file 41408_2021_484_MOESM6_ESM.jpg]

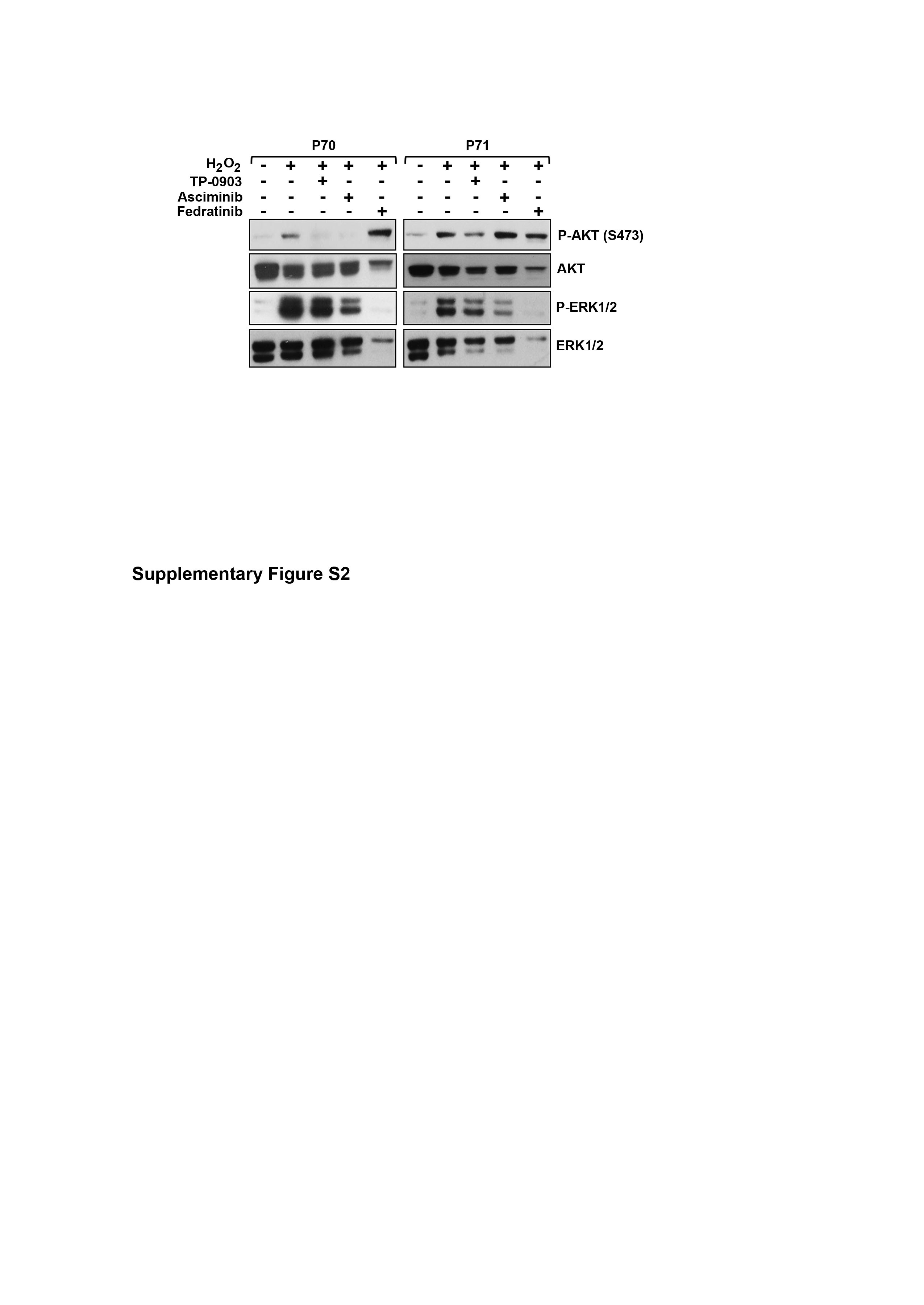

Supplement: Supplementary file 7 — Supplementary Figure S2 [file 41408_2021_484_MOESM7_ESM.jpg]

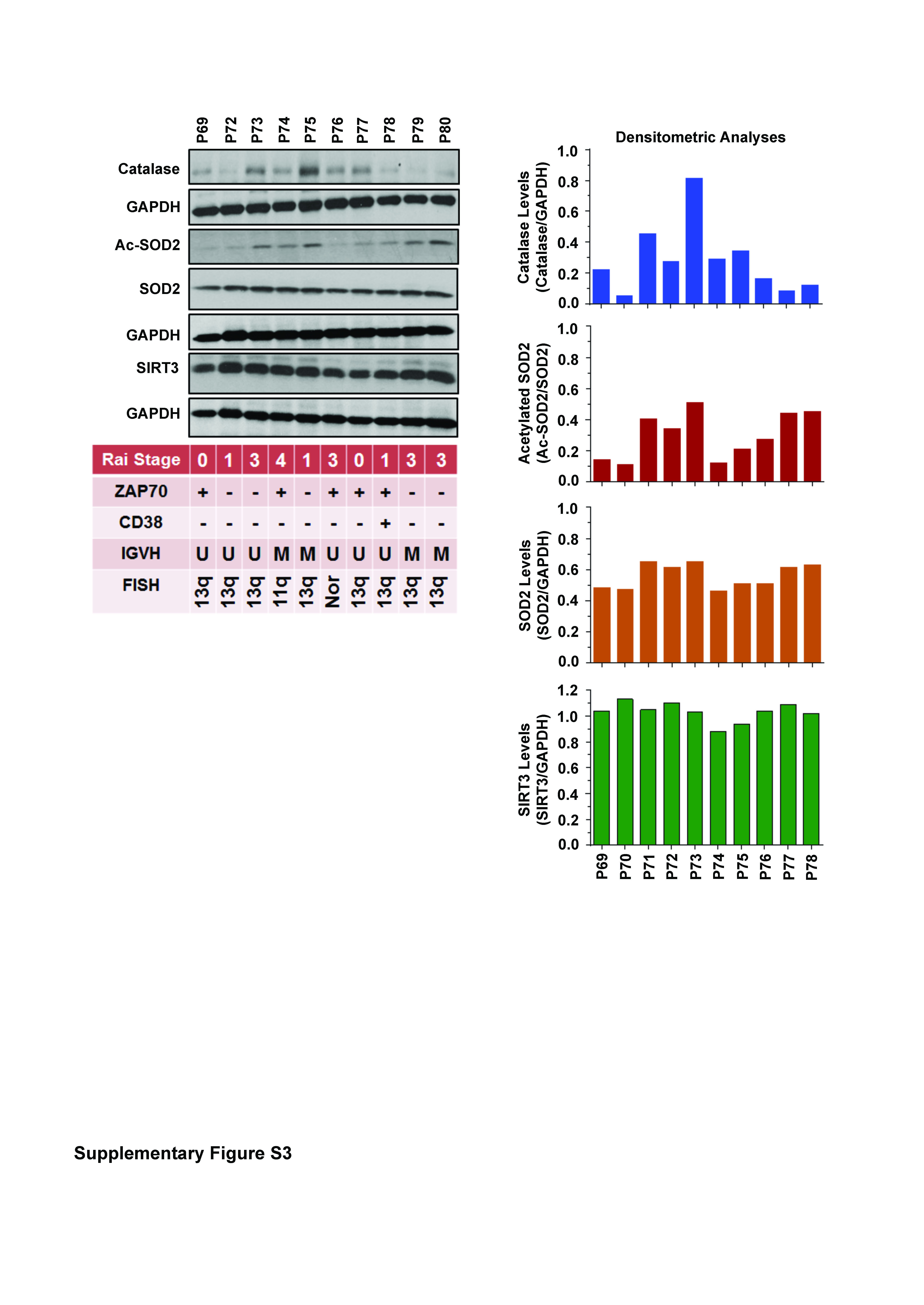

Supplement: Supplementary file 8 — Supplementary Figure S3 [file 41408_2021_484_MOESM8_ESM.jpg]

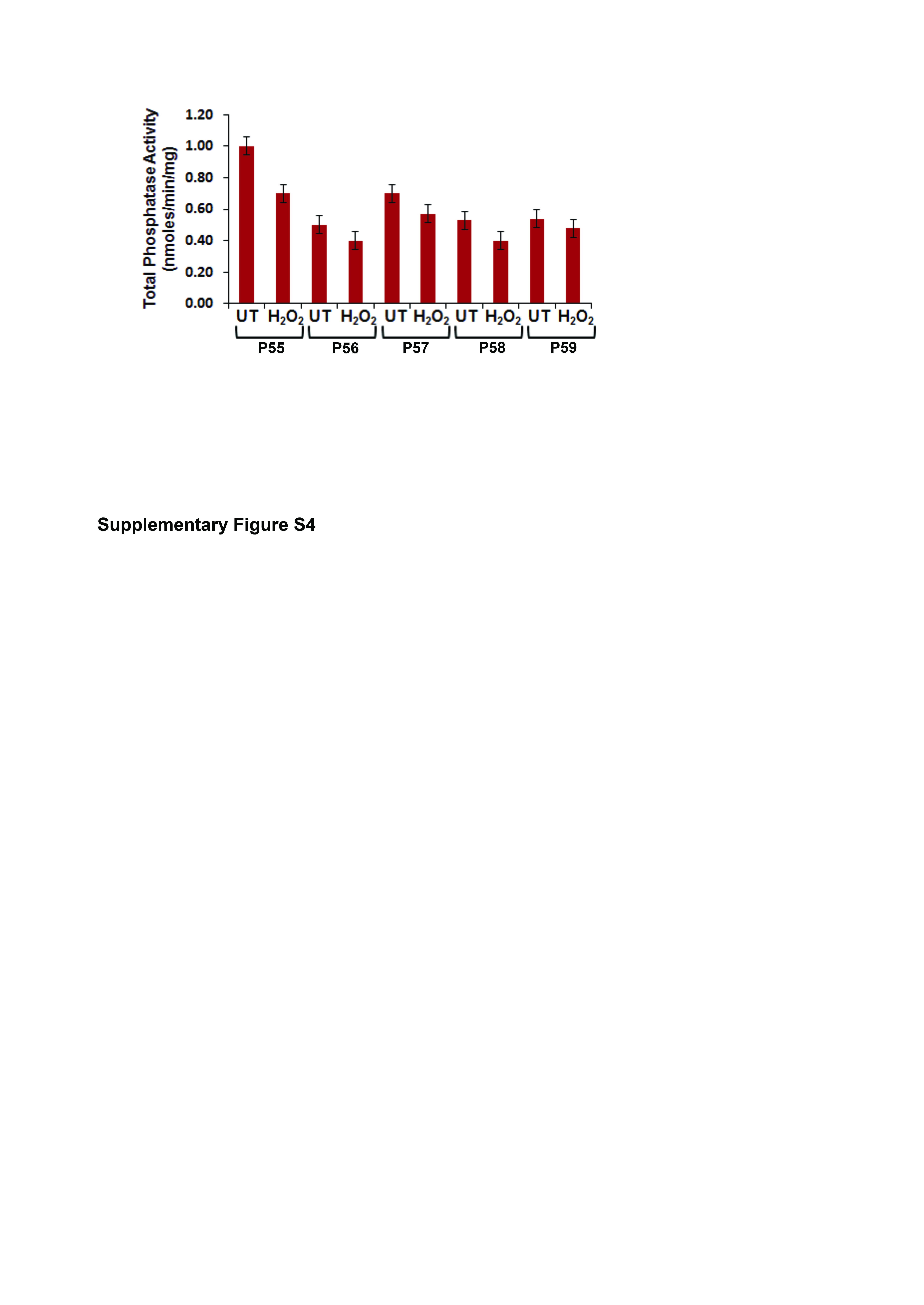

Supplement: Supplementary file 9 — Supplementary Figure S4 [file 41408_2021_484_MOESM9_ESM.jpg]
